# Formulation and Evaluation of Multilayered Tablets of Pioglitazone Hydrochloride and Metformin Hydrochloride

**DOI:** 10.1155/2014/848243

**Published:** 2014-05-12

**Authors:** Y. Ankamma Chowdary, Ramakrishna Raparla, Muramshetty Madhuri

**Affiliations:** ^1^Department of Pharmaceutics, NRI College of Pharmacy, Pothavarappadu Village, Krishna District, Andhra Pradesh 521 212, India; ^2^Department of Pharmaceutics, Vaageswari Institute of Pharmaceutical Sciences, Beside LMD Police Station, Karimnagar, Andhra Pradesh 505 481, India

## Abstract

In the treatment of type 2 diabetes mellitus a continuous therapy is required which is a more complex one. As in these patients there may be a defect in both insulin secretion and insulin action exists. Hence, the treatment depends on the pathophysiology and the disease state. In the present study, multilayered tablets of pioglitazone hydrochloride 15 mg and metformin hydrochloride 500 mg were prepared in an attempt for combination therapy for the treatment of type 2 diabetes mellitus. Pioglitazone HCl was formulated as immediate release layer to show immediate action by direct compression method using combination of superdisintegrants, namely, crospovidone and avicel PH 102. Crospovidone at 20% concentration showed good drug release profile at 2 hrs. Metformin HCl was formulated as controlled release layer to prolong the drug action by incorporating hydrophilic polymers such as HPMC K4M by direct compression method and guar gum by wet granulation method in order to sustain the drug release from the tablets and maintain its integrity so as to provide a suitable formulation. The multilayered tablets were prepared after carrying out the optimization of immediate release layer and were evaluated for various precompression and postcompression parameters. Formulation F13 showed 99.97% of pioglitazone release at 2 hrs in 0.1 N HCl and metformin showed 98.81% drug release at 10 hrs of dissolution in 6.8 pH phosphate buffer. The developed formulation is equivalent to innovator product in view of *in vitro* drug release profile. The results of all these evaluation tests are within the standards. The procedure followed for the formulation of these tablets was found to be reproducible and all the formulations were stable after accelerated stability studies. Hence, multilayered tablets of pioglitazone HCl and metformin HCl can be a better alternative way to conventional dosage forms.

## 1. Introduction

A drug delivery system (DDS) is defined as a formulation or a device that enables the introduction of a therapeutic substance in the body and improves its efficacy and safety by controlling the rate, time, and place of release of drugs in the body. The process includes the administration of the therapeutic product, the release of the active ingredient by the therapeutic product, and subsequent transport of the active ingredients across the biological membranes to the site of action. Oral route for the administration of therapeutic agents is the oldest and most convenient route because of low cost of therapy and ease of administration which leads to higher level of patient compliance. It has a wide acceptance up to 50–60% of total dosage forms and has been explored for systemic delivery of drugs via pharmaceutical products of different dosage form. Approximately 50% of the drug products available in the market are administered orally [1–3].

Conventional dosage forms are rapidly absorbed, with the ascending and descending portions of the concentrations versus time curve reflecting primarily the rate of absorption and elimination, respectively. Because of the rapid rate of absorption from conventional drug delivery systems, drugs are usually administered more than once daily, with the frequency being dependent on biological half-life (*t*(1/2)) and duration of pharmacological effect [4–6].

An appropriately designed controlled-release drug delivery system (CRDDS) can improve the therapeutic efficacy and safety of a drug by precise temporal and spatial placement in the body, thereby reducing both the size and number of doses required. The main objective of controlled/sustained release drug delivery is to make sure of safety and to improve effectiveness of drugs as well as patient compliance. Moreover, this controlled drug delivery system fails to achieve the stated advantages due to lack of releasing the initial bolus dose dumping and failure to achieve site specific drug delivery. Formulation of layers from different types of polymers allows manipulation over more than one rate-controlling polymer, thus enabling different kinds of drug delivery of one or more active ingredients, that is, where the drug may be released with a bolus and then at a controlled rate or by targeted drug delivery in the GI tract using pH dependent polymers [7, 8].

Diabetes mellitus is a chronic metabolic disorder characterized by a high blood glucose concentration, hyperglycemia (fasting plasma glucose >7.0 mmol/L, or plasma glucose >11.1 mmol/L two hours after a meal) caused by insulin deficiency, often combined with insulin resistance, where continuous therapy is required. Apart from diet and exercise, which are fundamental in the treatment of diabetes mellitus (DM), single oral antidiabetic drug sometimes fails to achieve and maintain glycemic control. Consequently, a combination therapy using agents with complementary mechanism of action has become a cornerstone of type 2 diabetes mellitus (T2DM) management [9].

Metformin HCl, the only available biguanide, remains the first line drug therapy for obese patients with T2DM. Daily dose of metformin HCl is 0.5–3 gm. In monotherapy under certain conditions, metformin at high doses can cause lactic acidosis, cataract. Pioglitazone HCl is an orally administered thiazolidinedione agent. Daily dose of pioglitazone HCl is 15–30 mg. Pioglitazone sensitizes peripheral tissues to insulin and hence may cause hypoglycemia when insulin is used concomitantly, though hypoglycemia can also occur with monotherapy. Multilayered tablets were formulated to avoid repetitive dosage form administration. Solubility of pioglitazone decreases with increase in pH and metformin is freely soluble drug, pioglitazone is formulated as immediate release layer, and metformin is formulated as controlled release layer. This type of combination will give better compliance [10–14].

The release retardant guar gum which was used in the present study is one of the most promising dietary fibers, which is a gel-forming galactomannan obtained by grinding the endosperm portion of* Cyamopsis tetragonolobus* L., a leguminous plant. All natural fiber diet works with body to achieve a feeling of fullness and to reduce hunger. It has also been used as an appetite suppressant [15–17].

## 2. Materials and Methods

### 2.1. Materials

Pioglitazone and metformin were obtained as a gift samples from Dr. Reddy's laboratories, Hyderabad and MSN formulations, Hyderabad, respectively, HPMC K4M and crospovidone were procured from Nihal traders, Hyderabad, guar gum is the grounded endosperm of* Cyamopsis tetragonolobus, *and all the remaining chemicals are of analytical grade.

### 2.2. Method

#### 2.2.1. Preparation of Immediate Release Layer of Pioglitazone HCl (F1–F4)

Immediate release layer of pioglitazone HCl was prepared by direct compression method (Figure 4). Pioglitazone HCl, crospovidone, avicel pH-102, and lactose were accurately weighed and passed through sieve number 40. All the above ingredients as shown in Table 1 were mixed in a polybag. Talc and magnesium stearate were added after passing through sieve number 40 and mixed properly.

#### 2.2.2. Preparation of Controlled Release Layer of Metformin HCl Formulated with HPMC K4M (F5–F8)

Controlled release layer of metformin HCl containing HPMC K4M was prepared by direct compression method. Metformin HCl, HPMC K4M, PVP K30, and dicalcium phosphate were passed through sieve number 40. All the above ingredients were mixed in a polybag. Talc and magnesium stearate were added after passing through sieve number 40 and mixed properly.

#### 2.2.3. Preparation of Controlled Release Layer of Metformin HCl Formulated with Guar Gum (F9–F12)

Metformin HCl granules containing guar gum were prepared by wet granulation technique by adding PVP K 30 dissolved in distilled water as a granulating fluid. Required quantities of metformin HCl, guar gum, and dicalcium phosphate were passed through sieve number 40 and were mixed thoroughly and a sufficient volume of granulating fluid was added slowly. After enough cohesiveness was obtained, the mass was passed through sieve number 12. The obtained granules were dried at 50°C in hot air oven till a constant weight was obtained (until dry). The dried granules were then passed through sieve number 40. Talc and magnesium stearate were added after passing through sieve number 40 and mixed properly.

### 2.3. Procedure

Optimization process is done for immediate release layer in order to select the composition to form a multilayered tablet. The first layer consists of immediate release and the second layer consists of controlled release. The first layer was placed in the die cavity which consists of immediate release layer and punched with low compression force and then the second layer was placed in the die cavity which consists of controlled release layer and allowed for punching and finally barrier layer containing 35 mg of ethyl cellulose was placed in the die cavity and compressed with maximum compression force in order to obtain multilayered tablets by using 12 mm punches of SAIMACH SMD 16 station tablet compression machine with an average hardness of 6–8 kg/cm^2^. Prior to compression, the granules were evaluated for several tests.

The optimized immediate release tablet F3 is formulated into multilayer tablet containing metformin HCl as controlled release layer with two different polymers.

## 3. Evaluation of Flow Properties

### 3.1. Precompression Flow Properties [18–20]

The granules of all formulations were evaluated for powder flow properties independently for both immediate and controlled release layers. The fixed funnel method was employed to measure the angle of repose. Bulk and tapped densities were determined by tapped density apparatus from which compressibility index and Hausner's ratio values were calculated. Drug content of granules was determined spectrophotometrically and the granules prepared from wet granulation method (F9–F13) were subjected to moisture content and loss on drying by using hot air oven.

## 4. Evaluation of Multilayer Tablets [21–24]

### 4.1. Thickness

The thickness of the tablets was determined by using vernier calipers. Randomly 10 tablets selected were used for determination of thickness that was expressed in mean ± SD and unit is mm.

### 4.2. Uniformity of Weight

The individual weight of 20 tablets was taken after selecting them randomly for weight variation. Then their average weight and their mean and standard deviationwere calculatedand compared with the standards. The weight of the tablet being made is measured to ensure that it contains predetermined amount of drug.

### 4.3. Hardness

Hardness is termed as the tablet crushing strength and it is the force required to break a tablet diametrically. Hardness of tablets was measured by selecting 5 tablets randomly and the hardness of each tablet was measured with Monsanto hardness tester. The hardness was noted. The hardness is usually measured in terms of kg/cm^2^.

### 4.4. Friability

The tablet friability is a measure of loss due to abrasion. The preweighed tablets were exposed to repeated shocks in Roche Friabilator in which they are initially weighed (*W*_*o*_) and kept in a tumbling and rotating apparatus drum and were subjected to fall from 6 inches height. After completion of 100 rotations, the tablets were reweighed (*w*) and the percent loss in weight or friability (*f*) was calculated by the formula given below:

(1) $$\begin{array}{c} \%\text{Friability}=\frac{\text{Initial}\;\text{weight}-\text{Final}\;\text{weight}}{\text{Initial}\;\text{weight}}\times 100. \end{array}$$

### 4.5. Disintegration Time

The disintegration time was determined at 37 ± 0.5°C using disintegration test apparatus in 0.1 N HCl.

### 4.6. Content Uniformity

Twenty tablets were powdered, and dose equivalent weight of powder blend was accurately weighed and transferred into a 100 mL volumetric flask. Initially 5 mL of methanol was added and shaken for 10 min. Then volume was made up to 100 mL with the methanol. The solution in the volumetric flask was filtered, diluted suitably, and analyzed spectrophotometrically. The drug content should be within the range between 90 and 110% of standard amount:

(2) $$\begin{array}{c} \%\text{Drug content}=\frac{\text{Drug content}}{\text{label}\;\text{claim}}\times 100. \end{array}$$

### 4.7. *In Vitro *Dissolution Studies

*In vitro* dissolution studies was conducted using USP dissolution apparatus-I at 37 ± 0.5°C temperature and at 50 rpm and the volume of dissolution media is 900 mL. 0.1 N hydrochloric acid was used as dissolution medium for the first two hours and 6.8 pH phosphate buffer for the remaining time. Samples of 5 mL were withdrawn at predetermined time intervals and replaced with 5 mL of fresh dissolution medium. The collected samples were suitably diluted with dissolution fluid, wherever necessary, and were analyzed for the pioglitazone HCl for the first two hours at 269 nm and for metformin HCl for the remaining time at 233 nm by using a double beam UV spectrophotometer.

### 4.8. Release Kinetics

The rate and mechanism of metformin release from the prepared multilayer tablets were analyzed by fitting the data in zero order as cumulative amount of drug release versus time:

(3) $$\begin{array}{c} C=K_{0}t, \end{array}$$

where *C* is the amount of drug released at time t and *K*_0_ is the release rate constant, first order as log cumulative percent of drug remaining versus time:

(4) $$\begin{array}{c} \text{Log}\;C=\text{Log}\;C_{0}-\frac{Kt}{2.303}, \end{array}$$

where *C*_0_ is the initial concentration of drug and *K* is the first-order rate constant, Higuchi model as cumulative percentage of drug released versus square root of time:

(5) $$\begin{array}{c} Q=Kt^{1/2}, \end{array}$$

where *Q* is the amount of drug released at time *t* and *K* is the diffusion rate constant, Korsmeyer-Peppas model as log time versus log cumulative percent drug release:

(6) $$\begin{array}{c} \text{Log}\left(\frac{M_{t}}{M_{\infty }}\right)=\text{Log}K+n\;\text{Log}\;t, \end{array}$$

where *M*_*t*_ is the amount of drug released at time *t*, *M*_*∞*_ is the amount of drug release after infinite time, *K* is release rate constant, and *n* is the diffusion exponent indicative of the mechanism of drug release.

### 4.9. Swelling Behavior of Tablets

The extent of swelling was measured in terms of  % weight gain by the tablet. The swelling behaviors of all controlled release formulations were studied. Initially one tablet from each formulation was kept in a petridish containing 6.8 pH phosphate buffers. The tablet was removed, lightly blotted with tissue paper to remove excess buffer, and reweighed for every 1 h; the weights of the tablet were noted. Percentage weight gain by the tablet was calculated by the following formula:

(7) $$\begin{array}{c} \text{S. I.}=\left\{\frac{\left(W_{t}-W_{0}\right)}{W_{0}}\right\}\times 100, \end{array}$$

where S.I. is the swelling index, *W*_*t*_ the weight of tablet at time *t* (h), and *W*_0_ the weight of tablet at zero time.

### 4.10. Stability Studies

The tablets were packed in suitable packaging and the well-sealed tablets were kept in the humidity chamber and their stability studies were conducted as per ICH and WHO guidelines to assess the drug content samples which were collected at the end of the studies and were evaluated for drug release and its content. The conditions prescribed by ICH guidelines for accelerated studies are 40°C ± 2°C/75% RH ± 5% RH for 3 months.

## 5. Results and Discussion

### 5.1. FTIR Studies

**Figure 1:**
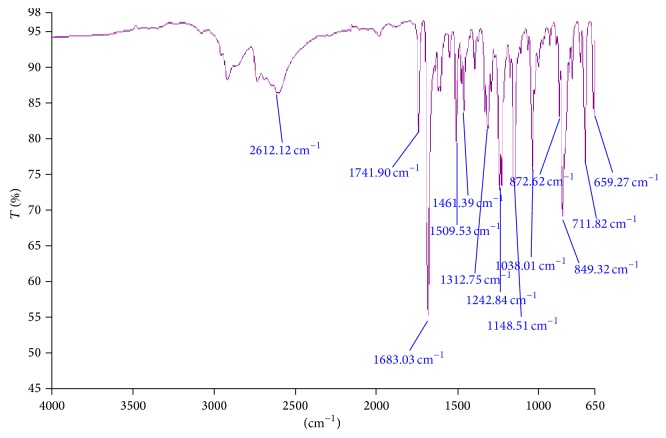
FTIR graph of pure drug pioglitazone HCl.

**Figure 2:**
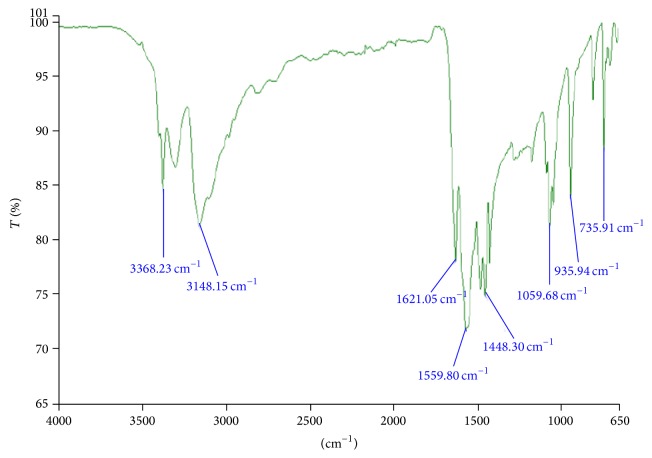
FTIR graph of pure drug metformin HCl.

**Figure 3:**
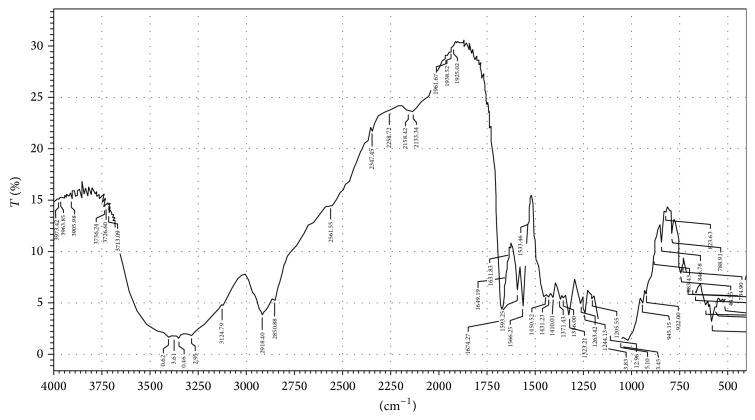
FTIR graph of optimized formulation.

**Figure 4:**
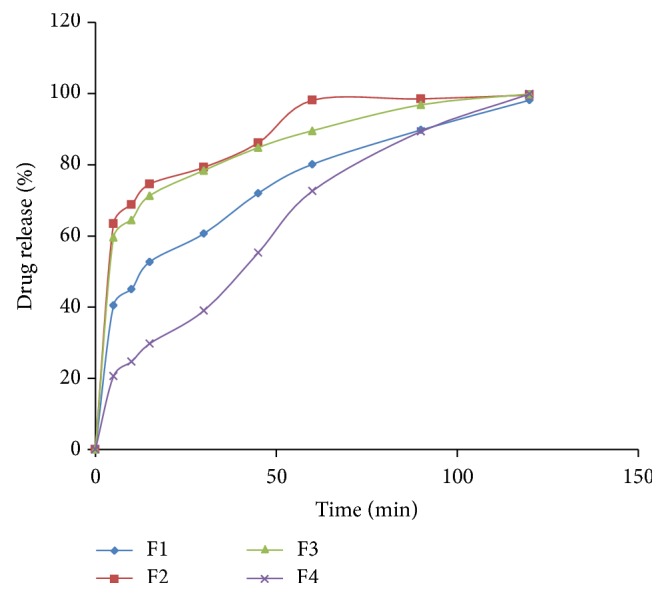
Dissolution graph of pioglitazone HCl immediate release tablets (F1–F4).

In order to investigate the possible interactions among pioglitazone HCl, metformin HCl, and different polymers/diluents, FTIR studies were carried out (Figures 1, 2, and 3). As the identical peaks were observed in all the cases, hence it was confirmed that no interaction exists between drugs and excipients. The characteristic peaks obtained in FTIR studies were shown in Table 3.

### 5.2. Precompression Flow Properties

All the formulations showed good flow properties with angle of repose values between 25° and 35° and Hausner's ratio ranged between 1.10 and 1.19, whereas Carr's index values ranged between 11 and 15, and all these values are shown in Tables 4 and 5.

### 5.3. Evaluation of Prepared Tablets

The tablets were visually observed and free from defects such as lamination, chipping, and capping. The prepared tablets passed all the in-process tests. The weights of the tablets are within the range. Hardness of the tablets was in the range of 2.9–7.3 kg/cm^2^, thickness of the tablets is within 2.65–5.20 mm, and the friability ranges between 0.17 and 0.81 percent. These results are shown in Tables 6 and 7.

### 5.4. *In Vitro *Drug Release Profile

The drug release from F1 composed of 10% crospovidone is 98.14 ± 0.3%, F2 composed of 15% crospovidone is 99.60 ± 0.1%, F3 composed of 20% crospovidone is 99.89 ± 0.4%, and F4 composed of 25% crospovidone is 99.86 ± 0.2%. The concentration of avicel PH 102 was kept at constant in all the formulations, that is, 15%. The drug release at specific time intervals was plotted in a graph and from the drug release profile formulation F3 composed of 20% crospovidone showed good drug release profile (99.97 ± 0.5%) with least disintegration time. Hence, F3 was selected as the optimized formulation for immediate release layer for incorporation with metformin controlled release layer. The drug release from crospovidone may be due to its capillary activity with the dissolution media [25].

**Figure 5:**
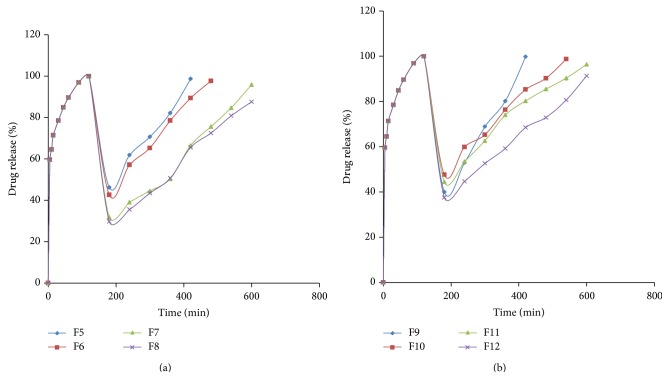
Dissolution graph of optimized immediate release layer formulated with metformin controlled release layer containing (a) HPMC K4 M (F5–F8) and (b) guar gum (F9–F12).

**Figure 6:**
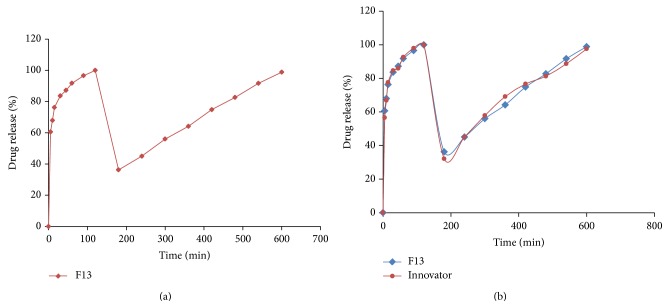
Dissolution graph of optimized guar gum formulation (F13) (a) individually and (b) compared with innovator.

The optimized F3 was formulated into bilayer tablets containing metformin controlled release layer along with it (Table 2). The drug release from F11 was better and a barrier layer was incorporated to it in F13 to release the drug in a controlled manner. The drug release profile of the tablets was shown in Figures 5 and 6.

The drug release of pioglitazone HCl from F13 was found to be 99.94 ± 0.7% at 2 hrs of dissolution in 0.1 N HCl. The drug release of metformin HCl from F13 was found to be 98.81 ± 0.5% at 10 hrs of dissolution in 6.8 pH phosphate buffer.

The release of drug not only depends upon the nature of matrix but also depends upon the drug polymer ratio. Due to the differences in the rheological properties of the polymers, they are taken in different concentrations. Guar gum at low concentrations (F9) can retard the drug release very well compared to HPMC K4M (F5) that can retard the drug release at a slight higher concentration compared to guar gum, whereas the drug retarding capacity is similar at higher concentrations for HPMC (F7 and F8) and guar gum (F11 and F12). As HPMC K4M is a semisynthetic polymer, by direct compression, high binder concentrations are required to maintain the tablet integrity due to the poor compressibility problems of metformin HCl compared to less concentrations of binder in wet granulation formulated with guar gum that acts as a good binding agent and maintains integrity of tablets up to 8 hrs of dissolution in 6.8 pH phosphate buffer. The filler dicalcium phosphate employed in the formulations (F5–F13) is insoluble and nonswelling and also slows the drug release from the matrix [26, 27].

HPMC forms low density hydrocolloid system and a hydrogel layer would be formed which acts as a gel boundary for the delivery system that retards the drug release. The tablets swelled radially and axially during the* in vitro* swelling studies of HPMC and the dissolution of drug can be diffusion controlled by the formation of gel that forms a viscous layer acting as a protective barrier to both the influx of water and the efflux of the drug in solution from the polymer matrix as water uptake increases with increase in concentration of polymer, whereas the penetration of water into tablets formulated with guar gum (F9–F13) was rather slow. The guar gum formulations showed an initial burst of drug release from the matrix-embedded system, diffusion coupled with erosion might be the mechanism of the drug release at low concentrations, and the slow drug release was observed with increase in the concentration of the polymer; this might be due to the formation of thick gel layer with increasing viscosity around the tablets and this gel further inhibits the entry of release medium into the matrix and control release of the drug. The reason behind this is due to the slow erosion of the gelled layer, backbone mechanism for the drug release from the tablets containing higher amounts of guar gum [28, 29].

### 5.5. Release Kinetics

The data suggested that the release kinetics of the drug follow zero order drug release, as the values of regression coefficient obtained for zero order drug release profiles (*r* ^2^ = 0.8524 to 0.9932) are higher as compared to first-order drug release profiles (*r* ^2^ = 0.0098–0.4866). The* in vitro* release profiles of drug from all these formulations could be best expressed by Higuchi's equation as the plots showed the highest linearity (*r* ^2^ = 0.9422 to 0.9966). This indicates that the drug release from the tablets was diffusion method. To confirm the exact mechanism of drug release further the data was fitted in Korsmeyer-Peppas equation and the results showed that the drug release follows diffusion mediated Fickian model from the *n* value (*n* < 0.45) (Table 8).

### 5.6. Swelling Studies

**Figure 7:**
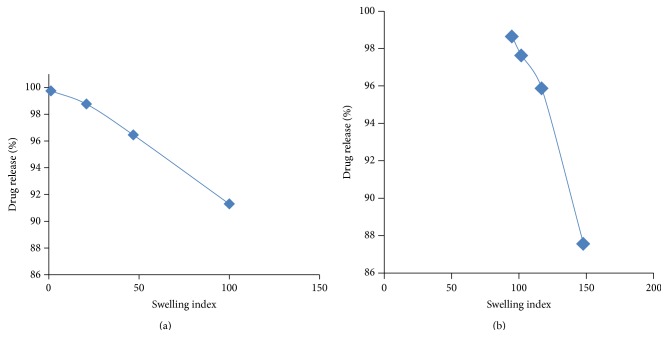
Graph of swelling index versus % drug release of tablets formulated with (a) HPMC K4 M and (b) guar gum.

The swelling index was found to be increased with the polymer concentration. The swelling index was found to be increased with polymer concentration. There was a good correlation between the swelling index and* in vitro* drug release (Figure 7).

### 5.7. Stability Studies

**Figure 8:**
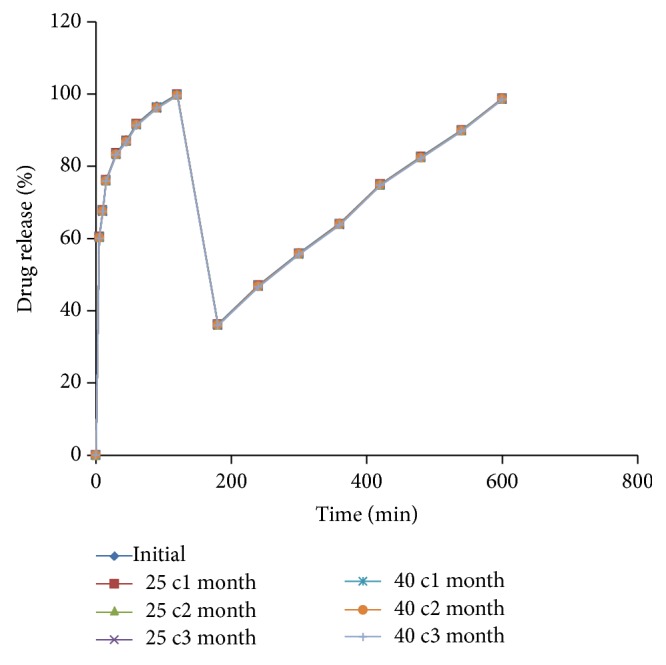
Stability studies graph of optimized multilayered tablets.

Accelerated stability studies were performed as per ICH guidelines for optimized formulation. The tablets showed no significant changes in the physical appearance and color. The tablets were evaluated at initial, 1st month, 2nd month, and 3rd month for* in vitro* drug release. The* in vitro* release profile was the same as that of the initial one and the results were shown in Figure 8, indicating that the formulation was quite suitable.

### 5.8. Comparison of Optimized Formulation with Marketed Formulation

The* in vitro* drug release profile of optimized formulation (F13) was compared with that of the marketed formulation (PIO-FIX MF 15). The % drug release of pioglitazone from marketed formulation was found to be 56.57 ± 0.3% at 5 min and 99.97 ± 0.5% at 2 hrs, whereas the drug release from the optimized formulation was found to be 60.57 ± 0.8% at 5 min and 99.94 ± 0.7% at 2 hrs, indicating that the drug release from the optimized formulation of immediate release layer of pioglitazone HCl showed better drug release compared to the innovator. The % drug release of metformin HCl from marketed formulation was found to be 97.5 ± 0.8% and from F13 was 98.81 ± 0.5% at 10 hrs. Hence, the drug release from optimized formulation was better when compared to marketed formulation.

## 6. Conclusion

From the experimental results, the following was concluded.

Pioglitazone HCl and metformin HCl along with the polymers and other excipients which are selected were compatible and were confirmed from the FTIR studies. A barrier layer is incorporated in F13 to release the drug in a controlled manner, such that the drug is to be eliminated before the administration of next dosage form, and in night times the metabolic processes are slow and the plasma drug concentration levels decline slowly due to the reduced elimination rate. The drug release rate from immediate layer was found to be dependent on the concentration of superdisintegrants employed whereas the controlled release layer was found to be dependent on the type of and polymer concentration employed in the tablet formulation. This may be due to the variations in the viscosity, swelling index, and type of release mechanism involved. The formulation F13 formulated with crospovidone-20%, avicel PH 102-15% in immediate release layer, guar gum-22.5%, and PVP K30-2% in controlled release layer along with the barrier layer showed the required drug release profile.

## Figures and Tables

**Table 1:** Composition of immediate release layer formulations of pioglitazone HCl.

Formulation code	F1	F2	F3	F4
Pioglitazone HCl (mg)	15	15	15	15
Crospovidone (mg)	1.5	2.25	3	3.75
Avicel PH 102 (mg)	2.25	2.25	2.25	2.25
Lactose (mg)	59.25	58.5	57.75	57
Magnesium stearate (mg)	1	1	1	1
Talc (mg)	1	1	1	1
Total weight (mg)	**80**	**80**	**80**	**80**

**Table 2:** Optimized immediate release layer along with controlled release layer is formulated as bilayer tablets.

Formulation code	F5	F6	F7	F8	F9	F10	F11	F12	F13
Immediate release layer									
Pioglitazone HCl (mg)	15	15	15	15	15	15	15	15	15
Crospovidone (mg)	3	3	3	3	3	3	3	3	3
Avicel PH 102 (mg)	2.25	2.25	2.25	2.25	2.25	2.25	2.25	2.25	2.25
Lactose (mg)	57.75	57.75	57.75	57.75	57.75	57.75	57.75	57.75	57.75
Magnesium stearate (mg)	1	1	1	1	1	1	1	1	1
Talc (mg)	1	1	1	1	1	1	1	1	1
Controlled release layer									
Metformin HCl (mg)	500	500	500	500	500	500	500	500	500
HPMC K4M (mg)	50	75	112.5	125	—	—	—	—	—
Guar gum (mg)	—	—	—	—	37.5	75	112.5	150	112.5
PVP K30 (mg)	25	25	25	25	10	10	10	12	10
Dicalcium phosphate (mg)	87	62	24.5	12	114.5	77	39.5	—	39.5
Magnesium stearate (mg)	4	4	4	4	4	4	4	4	4
Talc (mg)	4	4	4	4	4	4	4	4	4
Distilled water	—	—	—	—	Q.S	Q.S	Q.S	Q.S	Q.S
Barrier layer									
Ethyl cellulose (mg)	—	—	—	—	—	—	—	—	35
Total weight (mg)	**750**	**750**	**750**	**750**	**750**	**750**	**750**	**750**	**785**

**Table 3:** Characteristic peaks of FTIR graphs.

Bond and frequency (cm^−1^)	Pure drugs		Optimized multilayer tablet formulated with guar gum (cm^−1^)
	Metformin (cm^−1^)	Pioglitazone (cm^−1^)	
N–H (1°) 3400–3250	3368.23	—	3373.61

N–H (2°) 3400–3250	3148.15	—	3282.95

C=N 1471–1689	1559.80	—	1566.25

C=O 1760–1665	—	1741.90	1674.27

C–O 1000–1300	—	1242.84	1244.13

C–S 600–700	—	659.27	682.82

C=N 1471–1689	—	1509.53	1533.46

**Table 4:** Flow properties of granules prepared by direct compression method.

Batch number	Angle of repose	Bulk density (g/mL)	Tapped density (g/mL)	Compressibility index (%)	Hausner's ratio	Content uniformity (%)
F1	31.9 ± 0.03	0.63 ± 0.01	0.74 ± 0.04	11.9 ± 0.02	1.17 ± 0.05	98.8 ± 0.06
F2	34.5 ± 0.06	0.75 ± 0.08	0.89 ± 0.02	12.6 ± 0.04	1.18 ± 0.04	100.7 ± 0.02
F3	33.8 ± 0.08	0.65 ± 0.03	0.76 ± 0.07	14.4 ± 0.05	1.16 ± 0.06	98.5 ± 0.07
F4	32.6 ± 0.02	0.72 ± 0.01	0.81 ± 0.06	12.4 ± 0.06	1.12 ± 0.03	97.8 ± 0.04
F5	32.6 ± 0.05	0.43 ± 0.08	0.51 ± 0.02	14.8 ± 0.06	1.18 ± 0.06	94.8 ± 0.04
F6	33.8 ± 0.04	0.51 ± 0.04	0.59 ± 0.05	13.9 ± 0.03	1.15 ± 0.04	99.6 ± 0.03
F7	30.5 ± 0.06	0.46 ± 0.05	0.55 ± 0.08	11.4 ± 0.07	1.19 ± 0.01	97.9 ± 0.06
F8	34.7 ± 0.08	0.39 ± 0.01	0.46 ± 0.01	12.7 ± 0.04	1.17 ± 0.09	98.6 ± 0.01

**Table 5:** Flow properties of granules prepared by wet granulation method.

Batch number	Angle of repose	BD (g/mL)	TD (g/mL)	C.I (%)	Hausner's ratio	Content uniformity (%)	Moisture content (%)	Loss on drying (%)
F9	28.7 ± 0.02	0.56 ± 0.04	0.62 ± 0.09	11.8 ± 0.07	1.107 ± 0.07	102.6 ± 0.04	4.6 ± 0.6	6.2 ± 0.4
F10	26.3 ± 0.07	0.53 ± 0.08	0.59 ± 0.06	13.4 ± 0.04	1.113 ± 0.08	98.8 ± 0.05	5.1 ± 0.2	6.7 ± 0.1
F11	25.8 ± 0.06	0.59 ± 0.01	0.65 ± 0.05	12.6 ± 0.06	1.101 ± 0.05	101.6 ± 0.02	4.3 ± 0.5	6.5 ± 0.8
F12	29.1 ± 0.04	0.55 ± 0.05	0.61 ± 0.02	14.8 ± 0.09	1.109 ± 0.04	98.5 ± 0.05	5.8 ± 0.4	5.9 ± 0.3
F13	25.9 ± 0.08	0.57 ± 0.03	0.63 ± 0.07	12.3 ± 0.07	1.103 ± 0.04	99.8 ± 0.09	4.5 ± 0.7	6.4 ± 0.2

**Table 6:** Physical properties of immediate release tablets.

Batch number	Weight (mg)	Hardness (kg/cm^2^)	Thickness (mm)	Friability (%)	Disintegration time (min)	Content uniformity (%)
F1	78.2 ± 0.5	3.4 ± 0.05	2.65 ± 0.04	0.75 ± 0.01	19′36′′	100.6 ± 0.4
F2	81.6 ± 0.8	2.9 ± 0.13	2.68 ± 0.01	0.64 ± 0.05	1′24′′	98.6 ± 0.8
F3	79.6 ± 0.7	3.1 ± 0.08	2.67 ± 0.02	0.81 ± 0.03	16′12′′	99.1 ± 0.5
F4	80.3 ± 0.2	3.2 ± 0.02	2.68 ± 0.03	0.68 ± 0.04	16′30′′	97.3 ± 0.7

**Table 7:** Physical properties of multilayer tablets.

Batch number	Weight (mg)	Hardness (kg/cm^2^)	Thickness (mm)	Friability (%)
F5	749.7 ± 0.5	6.5 ± 0.01	5.08 ± 0.02	0.25 ± 0.03
F6	750.2 ± 0.2	5.9 ± 0.03	5.11 ± 0.01	0.38 ± 0.04
F7	751.0 ± 0.1	6.8 ± 0.02	5.09 ± 0.04	0.17 ± 0.02
F8	748.9 ± 0.3	6.7 ± 0.05	5.11 ± 0.02	0.36 ± 0.07
F9	749.5 ± 0.4	5.7 ± 0.04	5.07 ± 0.02	0.41 ± 0.05
F10	751.6 ± 0.1	6.8 ± 0.01	5.10 ± 0.03	0.28 ± 0.06
F11	750.1 ± 0.2	7.2 ± 0.03	5.09 ± 0.01	0.24 ± 0.03
F12	749.8 ± 0.4	6.9 ± 0.02	5.11 ± 0.06	0.36 ± 0.04
F13	785.8 ± 0.4	6.8 ± 0.04	5.20 ± 0.05	0.43 ± 0.02

**Table 8:** Release kinetics of metformin HCl controlled release layer (F5–F13).

Batch number	Zero order		Higuchi		Korsmeyer-Peppas model			First order	
	*r* ^2^	*k*	*r* ^2^	*k*	*r* ^2^	*k*-*a*	*n*	*r* ^2^	*k*
F5	0.9018	17.42	0.9924	42.15	0.3179	1.995	0.300	0.0098	0.0806
F6	0.9075	14.56	0.9966	39.21	0.1962	1.5995	0.204	0.0362	0.1105
F7	0.9533	10.28	0.9681	31.57	0.4287	1.8407	0.265	0.4647	0.2118
F8	0.9594	9.99	0.9749	30.69	0.4556	1.8155	0.259	0.4866	0.1865
F9	0.9511	18.14	0.9851	42.59	0.2937	1.9319	0.286	0.0138	0.1381
F10	0.8524	11.80	0.9856	35.75	0.0456	1.2217	0.087	0.0698	0.1450
F11	0.9062	10.30	0.9960	32.93	0.1318	1.1825	0.120	0.3679	0.2533
F12	0.9044	9.44	0.9878	30.09	0.2408	1.4256	0.154	0.4158	0.1888
F13	0.9932	9.66	0.9525	31.25	0.1307	1.6749	0.224	0.3698	0.2809
